# Testing chemotherapy efficacy in HER2 negative breast cancer using patient-derived spheroids

**DOI:** 10.1186/s12967-016-0855-3

**Published:** 2016-05-03

**Authors:** Kathrin Halfter, Oliver Hoffmann, Nina Ditsch, Mareike Ahne, Frank Arnold, Stefan Paepke, Dieter Grab, Ingo Bauerfeind, Barbara Mayer

**Affiliations:** SpheroTec GmbH, Am Klopferspitz 19, 82152 Martinsried, Germany; Department of Obstetrics and Gynecology, Hospital of the University of Munich, Marchioninistr. 15, 81377 Munich, Germany; Department of General, Visceral, and Transplantation Surgery, Hospital of the LMU Munich, Marchioninistr. 15, 81377 Munich, Germany; Department of Gynecology and Obstetrics, Technical University Munich, Ismaninger Str. 22, 81675 Munich, Germany; Klinikum Harlaching, Sanatoriumsplatz 2, 81545 Munich, Germany; Klinikum Landshut, Robert-Koch-Str. 1, 8434 Landshut, Germany

**Keywords:** Breast cancer, Personalized medicine, Preclinical treatment selection, In vitro diagnostics, Spheroid cell culture

## Abstract

**Background:**

Targeted anti-HER2 therapy has greatly improved the prognosis for many breast cancer patients. However, treatment for HER2 negative disease is currently still selected from a multitude of untargeted chemotherapeutic treatment options. A predictive test was developed using patient-derived spheroids to identify the most effective therapy for patients with HER2 negative breast cancer of all stages, for clinically relevant subgroups, as well as individual patients.

**Methods:**

Tumor samples from 120 HER2 negative patients obtained through biopsy or surgical excision were tested in the breast cancer spheroid model using scaffold-free cell culture. Similarly, spheroids were also generated from established HER2 negative breast cancer cell lines T-47D, MCF7, HCC1143, and HCC1937 to compare treatment efficacy of heterogeneous cell populations from patient tumor tissue with homogeneous cell lines. Spheroids were treated in vitro with guideline-recommended compounds. Treatment mediated impact on cell survival was subsequently quantified using an ATP assay.

**Results:**

Differences were observed in the metabolic activity of the untreated spheroids, whereby cell lines consistently achieved higher values compared to tissue spheroids (p < 0.001). A higher number of cells per spheroid correlated with a higher basal metabolic activity in tissue-derived spheroids (p < 0.01), while the opposite was observed for cell line spheroids (p < 0.01). Recurrent tumors showed a higher mean vitality (p < 0.01) compared to primary tumors. Except for taxanes, treatment efficacy for most tested compounds differed significantly between breast cancer tissue spheroids and breast cancer cell lines. Overall a high variability in treatment response in vitro was seen in the tissue spheroids regardless of the tested substances. A greater response to anthracycline/docetaxel was observed for hormone receptor negative samples (p < 0.01). A higher response to 5-FU (p < 0.01) and anthracycline (p < 0.05) was seen in high grade tumors. Smaller tumor size and negative lymph node status were both associated with a higher treatment efficacy to anthracycline treatment combined with 5-FU (cT1/2 vs cT3/4, p = 0.035, cN+ vs cN−, p < 0.05).

**Conclusions:**

The tissue spheroid model reflects current guideline treatment recommendations for HER2 negative breast cancer, whereas tested cell lines did not. This model represents a unique diagnostic method to select the most effective therapy out of several equivalent treatment options.

## Background

Although prognosis has vastly improved for HER2 positive breast cancer due to targeted anti-HER2 treatment options, the remaining majority of patients with HER2 negative disease lack directed therapy options. For these patients a number of guideline directed chemotherapy substances and regimen are available. Patients are stratified to a chemotherapy regimen according to routine clinicopathological criteria such as TNM-stage, hormone receptor status, grading and tumor histology, as well as prior medical history and existing comorbidities [1, 2]. This may result in an unfavorable prognosis, especially for patients not responding to treatment or with recurrent, metastatic disease, depending on the publication recurrence rate of approximately 30 % have been reported [3, 4].

Predictive tests or assays to direct cancer treatment decisions remain an unmet need in oncology diagnostics and in the field of personalized medicine [5–7]. As of today no method to predict chemotherapy outcome for individual breast cancer patients is recommended by all applicable international authorities for clinical utility [8]. Determining treatment success in the adjuvant and metastatic treatment setting is a process requiring a lengthy follow-up observation period, and a direct association is only possible through close surveillance [9, 10]. Clinical prognostic factors at diagnosis according to national and international guidelines are age, axillary lymph node status, differentiation grade, hormone receptor and/or HER2 status, as response to chemotherapy or pathologic complete response (pCR) following neoadjuvant chemotherapy [11, 12]. Predictive factors for a pCR in neoadjuvant chemotherapy are high grade (G3) and a negative hormone receptor status [13, 14]. Young age at diagnosis is associated with both pCR and overall survival [15].

Biomarkers currently being studied in clinical trials mostly focus on the molecular profiles which characterize the odds of recurrence [16–21]. Depending on the individual prognostic risk profile, a recommendation can be made if a chemotherapy in general would be beneficial or not, however a specific chemotherapy regimen selection currently cannot be made [22].

Out of the newer techniques such as tumor infiltrating lymphocytes, Ki-67, circulating tumor cells, genetic profiles, and PIK3CA analysis, only the uPA/PAI1 analysis as well as the 21-Gene-Recurrence Score have been validated in prospective clinical trials, however, only for specific clinical breast cancer subtypes in primary disease [22–25].

Laboratory models such as genetically engineered and patient-derived xenograft mouse models allow the study of deregulated pathways in the development of invasive tumors and the treatment efficacy in a living organism [26]. However, it has been criticized that these models do not accurately mimic tumorigenesis and metastasis in humans due to the influence of the murine microenvironment [27–29]. Other drawbacks of this approach are the time consuming and highly care intensive breeding and caretaking of these specifically bred immunodeficient mice, and the fact that preclinical drug testing often takes months until treatment effects can be determined [30, 31]. Another aspect is the cell heterogeneity of the human tumor which may not be fully represented in murine models [31, 32].

Breast cancer cell lines are another frequently used model system and have previously been closely associated to the originating patient tumors regarding genomic aberrations, protein expression even after prolonged culture, and treatment efficacy [33–35]. However, the heterogeneity of all the cell types found in patient tumors cannot be replicated using cell lines. A higher level of amplification and a differential protein expression pattern compared to primary tumor cells has also been observed in breast cancer cell lines, one explanation might be the source of the cells which is mainly from pleural effusions or triple negative primary cancer [36, 37].

One main criticism of standard 2D cell culture is the absence of the surrounding cells and microenvironment which have an important influence on tumor development and progression [35] and drug response [38]. Data suggests that these factors can be compensated by using 3D cell culture models [39]. The 3D culture models that are currently being investigated are organotypic explant cultures, polarized epithelial cell culture, artificial skin, microcarrier culture, and cellular spheroids [40, 41]. Although it is also possible to preserve intact human tissue in its 3D form such as in organotypic explant cultures, a simplified method is to generate 3D cell spheroids from cell lines or primary tumor cells [42, 43]. In comparison to 2D monolayer cell culture, the 3D models offer the advantage of a more tissue-like complexity and heterogeneity, similar cellular polarity and cellular interactions [44, 45]. The 3D architecture also results in the formation of a penetration barrier, much like in a patient tumor [46, 47]. Cancer cells grown in 3D have demonstrated similar behavior, structure, and organization compared to in vivo tissue [48–50]. In a previous study the spheroid model detailed herein has been proven to correctly predict treatment outcome for patients undergoing neoadjuvant chemotherapy for primary breast cancer [51].

The aim of this study is to determine the treatment efficacy of the most frequently applied treatment options on tumor tissue-derived spheroids from HER2 negative breast cancer patients. These treatment results were compared to those obtained from established HER2 negative breast cancer cell lines, namely MCF7, T-47D, HCC1143, and HCC1937. Clinical subgroups were analyzed separately to determine if groups of patients or individual patients could be identified which deviate from the mean in regard to treatment efficacy in vitro, thus determining new treatment strategies for these patients.

## Methods

### Patients and study design

The tissue was provided by a representative patient cohort of 120 HER2 negative breast cancer patients between 2011 and 2015 out of a total of 200 collected. Samples were considered in this analysis if patients were histologically confirmed HER2 negative (HER2+ , n = 45) and a valid assay readout was available (no readout, n = 43), a total of 8 samples failed to fulfill both criteria. Tissue samples were consecutively collected either through an ultrasound-guided core biopsy procedure (n = 77) or through surgical excision (n = 43). Tissue samples derived from primary (n = 109) and recurrent tumors (n = 11). Tissue from the recurrent tumors was obtained from local recurrence (n = 6), lymph nodes (n = 2), or distant metastasis (n = 3). Samples from two different tumor sites were obtained from five patients, in two cases from the originating breast tumor and the simultaneous lymph node metastasis, one case of invasive lobular carcinoma obtained from the right and left breast, as well as tumor tissue from two different metastatic sites from two patients. Clinical and pathological data is reported from the time of tissue excision. A written and oral informed consent was obtained from all patients prior to tissue excision, and an approval by all applicable ethics committees was obtained prior to study start.

### Breast cancer spheroid model

All laboratory procedures were performed according to standardized, quality-controlled operating procedures. The mean cold ischemic time for the surgical samples was 22.13 min (range 0–60 min), the time from tissue excision to the beginning of the cell isolation procedure was mean 24.09 h (range 14.75–48 h). Biopsy samples were directly transferred to freshly prepared culture medium following excision. Breast cancer spheroids were directly generated from cancerous tissue as described recently [51]. Briefly, fresh tumor tissue samples were mechanically and enzymatically (Roche, Germany) digested to generate a single-cell suspension. Cell number and viability of the single-cell suspension were determined using the trypan-blue exclusion test (Sigma Aldrich, Germany). The isolated cells were combined with a scaffold-free cell culture substrate and distributed evenly on a 96-well plate for subsequent spheroid formation.

Cell line spheroids were generated using the representative hormone receptor positive breast cancer cell lines MCF7 (HTB-22) [52], T-47D (HTB-133) [53], as well as the triple negative cell lines HCC1143 (CRL-2321) [54] and HCC1937 (CRL-2336) [55], all acquired from ATCC^®^ (American Type Culture Collection, USA). Cells were grown in appropriate culture medium and kept in culture for a maximum of 10 passages. For spheroid preparation, cells were detached using 1 mM EDTA and seeded 0.05 × 10^6^ per well. All experiments were repeated a minimum of three times, and given values represent a mean of all experiments with exceptions specifically detailed. The last passage of all four cell lines used in the treatment experiments was sent for external STR-analysis for authentication (IDEXX BioResearch, Germany). In addition cytospin samples of each cell line were prepared and stained using standard immunohistochemistry for confirmation of the epithelial origin, proliferation rate, and receptor status (data not shown).

Both primary tissue and cell line spheroids were kept under standard culture conditions (37 °C, 5 % CO_2_) for a consecutive 48 h following seeding. The spheroids were then treated with guideline recommended cytostatic compounds or combination of compounds using the peak plasma concentrations as detailed in a previous publication [1, 51, 56, 57]. Solvent controls were also run with each tissue and cell line experiment. The drug treatment was allowed to incubate for another 96 h. Treatment efficacy was assessed using an ATP assay (Promega, Germany) to quantify cell survival in vitro. Mean cell survival was expressed as percent of residual metabolic activity relative to the solvent controls. Analysis of tissue and cellular characteristics was done using all received tissue samples (n = 125), obtained from 120 patients.

### Statistics

Data comparison across cell lines and patients was done using the Kruskal–Wallis test and subsequent post hoc analysis; p-values were adjusted for multiple testing. Two-group comparisons were done using the Mann–Whitney U test. Bivariate correlation was done using the Pearson correlation procedure. Results were considered significant if the p value was lower than 0.05. All statistical analysis was done using IBM SPSS Statistics Version 22. The treatment efficacy heatmap was generated using the program R version 3.1.2, module gplots heatmap2.

## Results

### Patient characteristics

The mean age of the patients was 58 (range 21–85) years at sample excision. The majority of tumors were either ER and/or PR positive (66.8 %), stage cT1/2 (74.7 %), node negative (52.0 %), with a high-grade (50.2 %) invasive ductal/other histology (84.2 %). A mean Ki67 score of 39.22 % positive cells (range 0.01–100 %) was recorded for the collected study samples. Patient characteristics are summarized in Table 1.

**Table 1:** Patient cohort description

Characteristics	All HER negative patients	
	N	%
All Patients	120	–
*Tissue*		
Biopsy	77	61.8
Surgical	43	38.2
*Disease*		
Primary	109	91.1
Recurrent	11	8.9
*Age [years]*		
<50	39	24.0
≥50	81	76.0
*cT stage*		
T1/T2	70	74.7
T3/4	24	25.3
Not documented	26	–
*cN status*		
N+	49	48.0
N−	53	52.0
Not documented	18	–
*cM*		
M0	100	87.8
M1	15	12.2
Not documented	5	–
*Histology*		
Invasive ductal/other	101	84.2
Invasive lobular	17	15.8
Not documented	2	–
*Grading*		
G1/2	56	49.8
G3	57	50.2
Not documented	7	–
*Hormone receptor status*		
Positive	79	66.8
Negative	41	33.2
*ER*		
Positive	78	66.4
Negative	41	33.6
Not documented	1	–
*PR*		
Positive	69	59.0
Negative	50	41.0
Not documented	1	–
*Ki67*		
Mean (% positive stained cells)		39.22

Clinical data was collected at the time of tissue accruement. Histological data was taken from the pathological examination of the respective biopsy/surgical tissue. Hormone receptor status was considered positive if one or both ER and PR were found positive according to current guidelines for pathological examination, ≥1 % positive cells

*ER* estrogen receptor, *PR* progesterone receptor

### Comparison of baseline characteristics between tissue and cell line spheroids

Mean cell viability of single cell suspensions following cell isolation from fresh tumor tissues was 87.0 % (range 26.2–100 %). No differences in cellular viability was found for cells isolated from biopsy or surgical tissue (mean 87.6 biopsy vs. 85.9 % surgical samples). Differences in viability were detected between primary and recurrent tumors, the measured viability was higher for the recurrent tumor samples (mean 86.1 primary vs 93.5 % viability recurrent tumors, p < 0.001).

The number of isolated cells was highly dependent on the amount of tissue that was provided (p < 0.001). The surgical tissue weighed a mean of 1020.6 mg (range 58.6–7232.3 mg) and the biopsy samples a mean of 86.3 mg (range 10.5–513.3 mg). No significant difference in the number of cells per mg tissue was observed between surgical specimen 5201.1 cells/mg (range 284.5–58949.8 cells/mg) and biopsy samples 6061.0 cells/mg (range 78.18–84143.6 cells/mg; surgical specimen vs biopsy) or between primary (mean 4691.5, range 78.2–66571.4 cells/mg) and recurrent tumors (mean 14919.0, range 433.2–84143.6 cells/mg, primary vs recurrent tumors). However, due to the higher total tissue weight, a higher number of treatment options could be tested using surgical specimen: a mean of 8 (range 1–23) for the surgical specimen and 2 treatment options (range 1–9) for the biopsy samples (surgical vs biopsy samples, p < 0.001). Using regression analysis over all samples, it was determined that a minimum of 69 mg of tissue is required to test a minimum of three different treatment options (p < 0.001).

The amount of ATP quantified for solvent control samples (counts per second, cps) in the assay was measured to determine any differences in metabolic activity not associated with cytostatic treatment. Comparison of the tissue-derived spheroids revealed differences between biopsy and surgical samples in metabolic activity, biopsy samples reaching a mean of 0.479 cps/cell compared to a mean of 1.055 cps/cell for surgical samples (surgical vs biopsy samples, p < 0.01). The mean cps value relative to the number of cells per spheroid of the primary tumor spheroids was 0.628 cps; recurrent tumors achieved higher values with a mean of 1.022 cps, the difference was however not statistically significant. Spheroids generated from breast cancer cell lines showed much higher metabolic activity values with a mean of 26.55 cps (primary tumor samples vs. cell lines, p < 0.001, recurrent tumor samples vs. cell lines, p < 0.001). The highest values of metabolic activity was observed for MCF7 (36.85 cps/cell), followed by T-47D (32.36 cps/cell), HCC1937 (28.10 cps/cell), and HCC1143 (3.76 cps/cell), the difference between the cell lines was not significant.

**Fig. 1:**
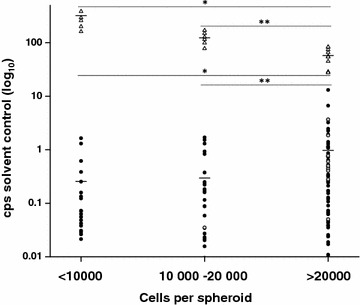
Metabolic activity of the spheroids (cps) measured after incubation with solvent control grouped according to cell number per spheroid. *Filled circle* primary tumor, *filled gray circle* recurrent tumor samples, *open triangle* cell lines, *line* mean, *p < 0.05, **p < 0.001. *cps* counts per second

In order to determine the effect of cell number in tissue-derived spheroids on metabolic activity data was collected using <10,000, 10,000–20,000, >20,000 cells per spheroid. As shown in Fig. 1, tissue-derived spheroids showed a higher metabolic activity the more cells were included per spheroid (p < 0.01). The difference in metabolic activity was significant between spheroids containing <10,000 (mean 0.256 cps) and >20,000 cells (mean 0.973 cps, p < 0.05). In addition, significant differences in metabolic activity were found between spheroids containing 10,000–20,000 cells (mean 0.296 cps) and >20,000 cells (mean 0.973 cps, p < 0.01). A correlation of metabolic activity with the absolute number of cells per spheroid was also significant, the more cells per generated spheroid resulted in a higher metabolic activity (p < 0.05). The results for the cell lines MCF7 and HCC1937 were inversely effected by the number of cells per spheroid: more cells resulted in a lower metabolic activity (p < 0.01). A correlation between cps and the proliferation index Ki67 was not detected.

### Treatment efficacy in tissue and cell line-derived spheroids

**Fig. 2:**
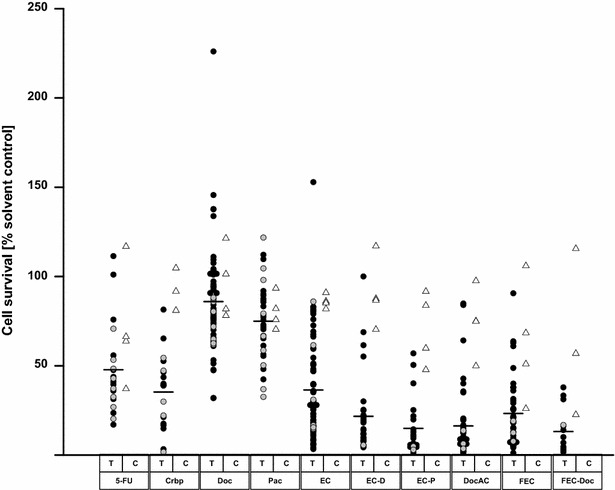
Dot plot grouped according to tested chemotherapy. *Filled circle* primary tumor, *filled gray circle* recurrent tumor samples, *open triangle* cell lines, *black line* mean T tissue, C cell *lines*. 5*-*FU 5-fluorouracil, Crbp carboplatin, Doc docetaxel, Pac paclitaxel, EC epirubicin + cyclophosphamide, DocAC docetaxel + doxorubicin + cyclophosphamide, FEC 5-fluorouracil + epirubicin + cyclophosphamide

Collectively, the tissue-derived spheroids showed a high variability in response to cytostatic treatment in vitro, regardless of primary or recurrent disease. Individual results are shown in Fig. 2. The best efficacy for primary tumor cell-derived spheroids was seen with FEC-Doc (5-FU + Epirubicin + Cyclophosphamide–Docetaxel) resulting in a mean of 13.62 % cell survival over all tested samples (n = 12), followed by EC-Pac (Epirubicin + Cyclophosphamide–Paclitaxel, 16.63 %, n = 19), and DocAC (Docetaxel + Doxorubicin + Cyclophosphamide, 17.37 %, n = 38, Table 2). Taxane treatment had the lowest impact on cell survival with a mean of 74.98 and 85.95 % for Pac and Doc respectively. Overall, combination treatments showed a better response compared to single-agent treatment for both primary and recurrent samples, carboplatin (Crbp) showing the best response in vitro (primary tumor samples, mean cell survival 36.97 %, n = 13, recurrent tumor samples, 31.07 %, n = 5). Generally the samples from recurrent tumors responded better to treatment in vitro as compared to samples from the primary tumors, although the differences were not statistically significant. For the recurrent tumor samples the best results were seen for treatment with EC-Pac (3.89 %, n = 2).

**Table 2:**
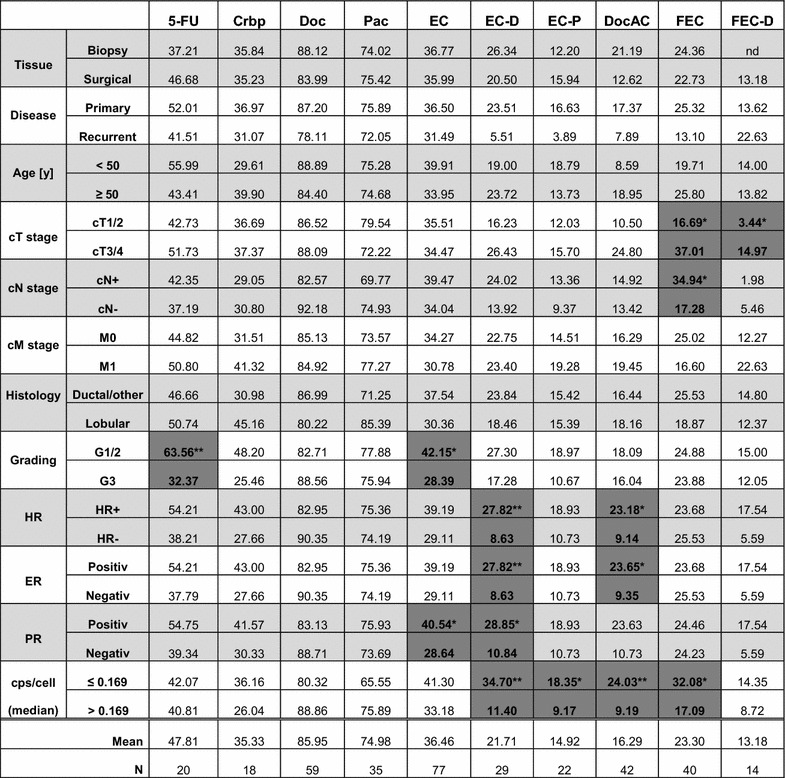
Treatment efficacy represented as cell survival relative to the respective solvent control

Mean values are shown in the breast cancer spheroid model for patient samples grouped according to clinical criteria, primary and recurrent tumors are included

Significant differences between clinical subgroups in treatment efficacy are highlighted in dark grey with bold font

* p < 0.05, ** p < 0.001

*HR* hormone receptor, *ER* estrogen receptor, *PR* progesterone receptor, *cps* counts per second, *5*-*FU* 5-fluorouracil, *Crbp* carboplatin, *Doc* docetaxel, *Pac* paclitaxel, *EC* epirubicin + cyclophosphamide, *DocAC* docetaxel + doxorubicin + cyclophosphamide, *FEC* 5-fluorouracil + epirubicin + cyclophosphamide, *nd* not done

Data shown in Fig. 2 demonstrated that cell line spheroids showed a consistently lesser cytostatic response than tissue spheroids. Exception was the taxane treatment, with Doc and Pac showing similar efficacy in both tissue and cell line spheroids. Individual treatment efficacy data for all cell lines is shown in Table 3.

**Table 3:** Table showing the mean cell survival as percent of the solvent control measured for the tested breast cancer cell lines according to the tested compounds ± standard deviation

Cell line	5-FU	Crbp	Doc	Pac	EC	EC-Doc	EC-Pac	DocAC	FEC	FEC-Doc
HCC1937	37.09 ± 1.92	80.95 ± 0.67	101.38 ± 2.69	93.37 ± 1.71	85.12 ± 5.26	87.57 ± 5.03	83.74 ± 10.44	75.06 ± 2.80	25.99 ± 4.12	22.56 ± 2.24
HCC1143	116.84 ± 13.72	104.71 ± 26.68	121.36 ± 45.60	81.97 ± 42.22	86.07 ± 24.99	117.00 ± 23.75	91.59 ± 33.35	97.56 ± 6.57	105.97 ± 20.15	115.57 ± 31.20
MCF7	63.77 ± 4.73	91.71 ± 13.55	81.80 ± 11.41	70.38 ± 5.01	90.96 ± 18.68	86.60 ± 15.68	59.78 ± 23.96	74.90 ± 16.61	68.40 ± 10.85	56.89 ± 13.37
T-47D	66.29 ± 2.38	nd	78.13 ± 7.33	75.83 ± 7.83	81.83 ± 7.13	70.33 ±	47.77 ± 8.98	49.88 ± 4.63	50.99 ± 10.40	nd
Mean	78.68	95.09	99.08	79.77	86.35	94.26	74.37	78.62	71.18	70.06

*HR* hormone receptor, *5*-*FU* 5-fluorouracil, *Crbp* carboplatin, *Doc* docetaxel, *Pac* paclitaxel, *EC* epirubicin + cyclophosphamide, *DocAC* docetaxel + doxorubicin + cyclophosphamide, *FEC* 5-fluorouracil + epirubicin + cyclophosphamide, *nd* not done

### Treatment efficacy for clinically relevant subgroups

In order to better identify patient subgroups responding best to individual treatment regimen, the results from the patient-derived spheroids were grouped according to the clinical factors reported in Table 1.

As shown in Table 2, smaller tumors responded better to treatment with FEC (cT1/2, n = 18, 16.69 % vs. cT3/4, n = 11, 37.01 % mean cell survival, p < 0.05), as well as FEC-Doc (cT1/2, n = 4, 3.44 % vs. cT3/4, n = 4, 14.97 % mean cell survival, p < 0.05). Similarly, a negative lymph node status was also associated with a better response to FEC (cN-, n = 17, 17.28 % vs. cN+ , n = 12, 34.94 % mean cell survival, p < 0.05). Grading was found to be significantly associated with EC treatment efficacy (p < 0.05), whereby G3 tumors (n = 29, 28.39 % mean cell survival) responded better than G1/2 tumors (n = 41, 42.15 % mean cell survival). A similar association was observed for the treatment with 5-FU (G3, n = 10, 32.37 % vs. G1/2, n = 8, 63.56, p < 0.01). The hormone negative subgroup showed higher treatment efficacy in response to EC-Doc (n = 7, 8.63 % mean cell survival, p < 0.01) and DocAC (n = 19, 9.14 %, p < 0.05) compared to the hormone receptor positive tumors [EC-Doc (n = 20, 27.82 % mean cell survival) and DocAC (n = 22, 23.18 %)]. Analysis of drug response in regard to the ER and PR status individually showed significant differences between positive and negative samples and remained robust for EC-Doc, not however for DocAC and EC although high differences in cell survival were evident. However, a total of 10 samples were ER+/PR− while only one sample was ER−/PR+. Differences in drug response were therefore observed in regard to EC and PR status: PR negative samples showed a higher response (n = 28, 28.64 % mean cell survival) compared to PR positive samples (n = 47, 40.54 %; p < 0.05). A difference in cytostatic efficacy in vitro was not detected between core needle and surgical tissue samples regardless of the tested substance. Age, nodal status, metastatic disease, Ki67, and tumor histology did not show a significant association with treatment response on tissue spheroids.

Treatment efficacy to Doc was greater with higher metabolic activity (p < 0.05). For all other tested compounds no significant correlation between these two factors was found. However, an effect was seen when comparing treatment efficacy with a metabolic activity above and below the observed median of 0.169 cps/cell (Table 2). Treatment efficacy was higher for samples with a metabolic activity above the median, the effect was seen for EC-Doc (>0.169 cps/cell, n = 16, 11.40 vs. ≤0.169 cps/cell, n = 9, 34.70 % mean cell survival, p < 0.01), EC-Pac (>0.169 cps/cell, n = 13, 9.17 vs. ≤0.169 cps/cell, n = 4, 18.35 % mean cell survival, p < 0.05), DocAC (>0.169 cps/cell, n = 24, 9.19 vs. ≤0.169 cps/cell, n = 16, 24.03 % mean cell survival, p < 0.01), as well as FEC (>0.169 cps/cell, n = 21, 17.09 vs. ≤0.169 cps/cell, n = 17, 32.08 % mean cell survival, p < 0.05).

Contrary data was found for the cell lines, both hormone receptor positive/low grade cell lines consistently responding better to treatment in vitro. This effect was seen for Doc (HR+/low-grade, 80.33 % vs. HR−/high-grade, 114.70 %, p < 0.05), EC-Pac (HR+/low-grade, 54.98 %, HR−/high-grade, 88.23 %, p < 0.05), DocAC (HR+/low-grade, 64.89 % vs. HR−/high-grade, 90.06 %, p = 0.028), and EC-Doc (HR+/low-grade, 80.09 % vs. HR−/high-grade, 104.39 %, p < 0.05).

A grouped comparison of receptor positive or negative tissue with the respective HR+ or HR− cell line spheroids showed no difference in the treatment with taxanes. However, for all other tested compounds drug efficacy was significantly higher in tissue spheroids regardless of hormone receptor status. Exception was the treatment efficacy for 5-FU resulting in a higher drug response in HR− tissue spheroids compared to HR− cell line spheroids (p < 0.05). Grouped comparison according to grading revealed tissue spheroids responding much better to treatment compared to the respective cell line spheroids with the same grading. Taxane treatment was not significantly more effective for either high- or low-grade tissue spheroids versus cell lines. Again, 5-FU was only significantly more effective for high-grade tissue spheroids compared to the matched cell lines (G3, p < 0.05).

### Treatment efficacy on an individual patient basis

**Fig. 3:**
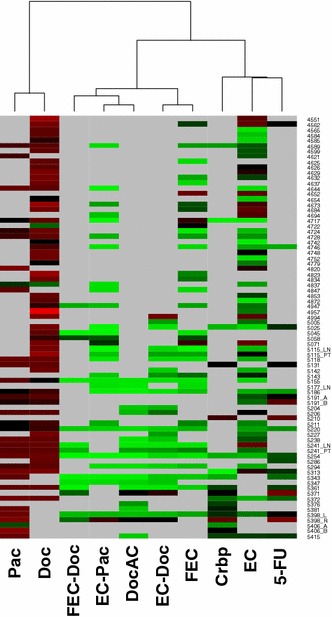
Heatmap comparing treatment efficacy for all individual patient samples where more than one drug/drug combination was tested. *Red colored boxes* show a poor (over 70 %), black a mean (from 35 to 70 %) and *green* a positive response to the applied chemotherapy in vitro (below 35 %). Simultaneous samples from one patient and different locations are indicated by additional *letters*. PT primary tumor, LN lymph node metastasis, L left, R, right, A, B, different metastatic sites

An overview of the assay data from all tested patients is shown in Fig. 3: red colored boxes show a poor (over 70 %), black a mean (from 35 to 70 %) and green a positive response to chemotherapy in vitro (below 35 %). The cutoff at 35 % was established in a previous study [51] and was therefore used again in this analysis; a mean cell survival above 70 % was considered resistant to cytostatic treatment in vitro and used as a second cutoff. The clustering of the data showed that taxanes as single-agents are the least effective anti-cancer drugs with only 2 out of 59 (3.4 %) for Doc and 1 out of 35 (2.9 %) for Pac achieving cell survival below 35 %. Also anthracyclines combined with taxanes are the most effective with 92.9 % of samples tested with FEC-Doc, 86.4 % with EC-Pac, 86.2 % with EC-Doc, and 85.7 % with DocAC reaching values below 35 % cell survival. The addition of 5-FU to the anthracycline-taxane combination therapy adds little benefit with 77.5 % of tested samples with FEC and 57.1 % of samples treated with EC showing a high response. Heterogeneous results were seen for Crbp treatment, out of the tested 18 samples only 8 (44.4 %) showed a high response in vitro.

**Fig. 4:**
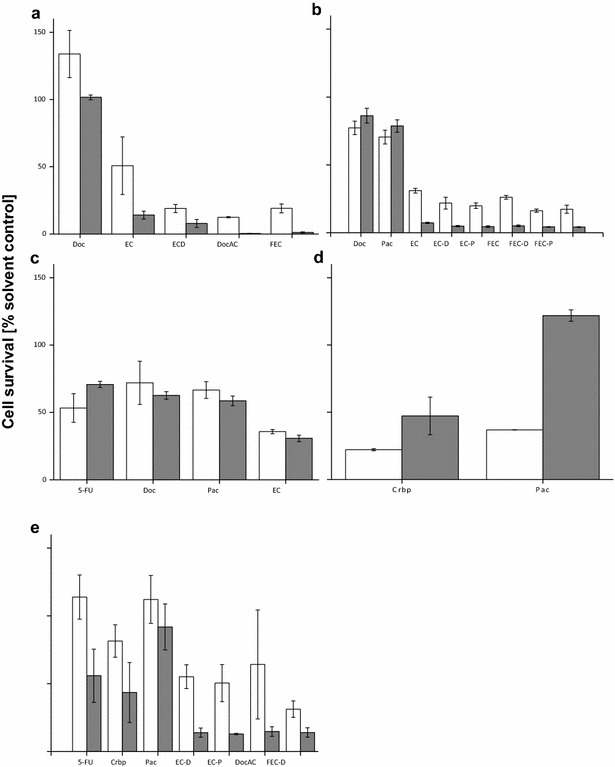
Individual patient cases where two tumor locations were compared according to in vitro treatment efficacy in the breast cancer spheroid model. **a**, **b** Patients number 5115 and 5241, comparison between simultaneous tumor of the breast (PT, *white*) and lymph node metastasis (LN, *gray*). **c**, **d** Show patients number 5191 and 5406, comparison of two different metastatic lesions in two cases of recurrent disease. **c** Shows two different metastasis of the peritoneal cavity, d were two metastatic lesions from the neck (*white bars*) and the shoulder (*gray*). **e** Shows patient number 5398, response pattern for a bilateral tumor in the *right* (*white bars*) and *left* (*gray*) breast

Detailed treatment options were further tested with spheroids generated from five different patients with disseminated tumors (Fig. 4). Results included comparison of drug efficacy of spheroids generated from primary tumors, simultaneous lymph node metastases and other metastatic sites, including peritoneal cavity, neck and shoulder, as well as one case of a bilateral tumor. As can be seen in these examples the different tumor locations obtained from an individual patient each show a differential response to in vitro treatment. Compared to the primary tumor, the lymph node showed a much better response in both of the tested patient samples. Similarly to the results seen over all patients, anthracycline-based treatment was most effective while taxane single compound treatment was not. Combined anthracycline-taxane treatment added to the cytostatic effect in vitro, although only slightly in both cases and respective tumor sites. The results from the different metastatic sites are not as consistent, the two metastatic sites within the peritoneal cavity showed similar treatment efficacy whereas the two locations from neck and shoulder did not. Due to the difference in tissue amount available not all treatment options could be tested. Treatment with EC showed the largest effect for the peritoneal metastasis while Crbp was most effective for the shoulder and neck metastasis. In the latter case a high discrepancy was seen in the response to Pac between the two metastatic sites, no cytostatic effect was observed for the shoulder metastasis while the tumor sample taken from the neck showed a marked response in vitro. The simultaneous tumors from the right and left breast also responded differently, the tumor from the left breast showing a higher treatment efficacy to all of the tested substances. Although standard deviation of the samples was very high, again the best response was seen for anthracycline-taxane treatment while Crbp showed the greatest effect of the single-compounds tested.

## Discussion

In this study, tissue-derived spheroids from HER2 negative patients were studied to determine differences in the observed treatment efficacy for the most frequently applied treatment schemes in the primary, recurrent, and metastatic setting and compared to results obtained from spheroids generated from HER2 negative breast cancer cell lines.

Regardless of the tested compound(s) and clinical characteristics, the range in treatment response for patient tissue-derived spheroids was highly heterogeneous. However, the mean values are representative of current international guideline recommendations [1, 56, 57]. Similarly, the association with clinically relevant subgroups with treatment efficacy in vitro reflects data found in largescale clinical trials or meta-analyses which favor an anthracycline-taxane based chemotherapy for HER2 negative patients. The addition of 5-FU to this combination has previously been found unbeneficial through large patient cohort analysis [58], this was also seen in the tissue spheroids tested in vitro. Comparison of the tested single compound agents showed that carboplatin was the most effective while 5-FU and both tested taxan compounds only had a small effect. Although the taxanes combined with an anthracycline were highly effective, similar to the high treatment success seen in the clinical application of this treatment combination.

Comparing the results obtained from tissue-derived spheroids according to hormone receptor status showed that a higher treatment efficacy to anthracycline-taxan combination treatment was found for hormone receptor negative tissue samples. This difference in response confirm results published by Kaufmann et al. [59] which also showed a similar in vitro resistance to Adriamycin for HR− primary breast cell culture. Interestingly, tissue spheroids recapitulate clinical findings that a triple negative tumor biology is associated with a high rate of pCR after chemotherapy [60]. An implicated mechanism for these observations was published recently by Lahsaee et al. [61]. Here, a reduced PRP4 K expression of the estrogen signaling pathway correlated with a reduced response to paclitaxel treatment. Similarly, high grade tissue spheroids consistently responded better to the anthracycline treatment. Surprisingly, treatment with 5-FU-based single- or combination treatment was found to be more effective for high-grade tissue samples, as well as to smaller tumors and node-negative patients. As mentioned above the use of 5-FU is under discussion, however distinct patient subgroups may profit from the application of this drug as an alternative to a taxane.

Other differences found in treatment efficacy between clinical subgroups were not statistically significant, mainly due to the large range in the collected data and the small sample size for individual subgroups. A fact reflecting this high heterogeneity in the treatment efficacy found for the tissue spheroids is that each patient tumor showed a distinct response pattern as seen in the heat map in Fig. 3, regardless of the tested treatment and clinical characteristics. Although it can be seen that individual patients did not follow this distinct pattern, here, other treatment combinations would have been more effective. Using this approach avoidable side effects might be prevented where two equivalent therapy options are identified, thus maximizing efficacy and minimizing toxicity. A similar effect was also found in two cases of primary breast tumor and the simultaneous lymph node metastases, two cases of separate metastatic sites and one case of bilateral lobular cancer despite having identical histopathological characteristics. The spheroids derived from the lymph node metastasis showed an overall better response to treatment than the primary tumor in both cases.

In general, cell line spheroids were consistently more resistant to in vitro treatment compared to tissue-derived spheroids. Treatment efficacy data was similar to other data previously reported in 3D cell culture [62, 63]. The differences between the two groups were apparent for most tested substances and combinations. Comparison of treatment efficacy according to hormone receptor status and grading showed contrary results to the tissue-derived spheroids, low grade and hormone receptor positive cell line spheroids responding better to cytostatic treatment in vitro. Reasons for this disparity might be that efficacy data from the breast cancer cell lines represent only a small range in the observed treatment efficacy found for the tissue-derived spheroids. This contrasting data might be due in part to the observed differences in baseline metabolic activity. The cell line spheroids showing contrary effects in regard to an increase in the number of cells per spheroid compared to tissue-derived spheroids. Another aspect might be the tissue composition: patient tissue-derived spheroids contain several different cell types not present in the cell line spheroids. Also it was observed that some cell lines do not show consistent results as reflected by high standard deviations for HCC1143 (Table 3), demonstrating that not all cell lines may be used as a standardized reproducible model.

Regarding the tissue-derived spheroids, the observed between patient, as well as intrapatient heterogeneity between tumor locations show the necessity for a more individualized treatment algorithm or diagnostic assay. The question remains for the ideal model to test treatment efficacy, currently no method is without its drawbacks, and however it is important to consider all aspects of tumor biology, as well as the tumor microenvironment [64, 65]. Current research on predictive biomarkers for breast cancer focuses mainly on a molecular and genomic characterization of patient tumors. Promising results have also been found in immunological biomarkers [66, 67]. However, although several biomarker studies have been conducted successfully using genetic or molecular analysis to predict treatment outcome, this approach has only been successfully validated for clinical routine in estrogen receptor positive, lymph node negative breast cancer patients [68–70]. Only these two methods were able to successfully add clinical predictive data to the established clinical characteristics [71]. Other predictive models each do not sufficiently represent the individual patient either through the influence of a murine microenvironment or through the insufficient representation of the patient tumor characteristics in vitro. The breast cancer spheroid model is representative in the 3D tumor heterogeneity and microenvironment and has been successfully associated with clinical treatment outcome in a previous study. An association between the data obtained through the breast cancer spheroid model and clinical treatment outcome i.e. pCR was observed [51]. In this analysis, tissue-derived spheroids from HER2 negative patients of all stages were studied to determine if any differences could be observed in regard to clinical subgroups and treatment response. This patient subgroup is currently still lacking directed treatment options and treatment outcome, and prognosis has not improved for this subgroup despite great achievements in tumor biology research and molecular characterization [72, 73].

Currently further data is necessary to determine an association with prognostic factors regarding survival and an additional interventional study to determine the independent prognostic and predictive power of the breast cancer spheroid model. Data obtained through these additional studies may aid in bridging the gap between guideline-directed treatment recommendations and tumor-directed therapeutic needs using a more representative in vitro model.

## Conclusion

The current study was conducted to provide additional data to validate the breast cancer spheroid model and its predictive potential regarding clinical treatment outcome for HER2 negative breast cancer patients. Tissue-derived spheroids treated in vitro with guideline recommended cytostatic drugs and drug combinations recapitulate clinical findings for HER2 negative patients. These findings are in stark contrast to HER2 negative breast cancer cell lines suggesting an impact of the stromal microenvironment in drug response. Taken together, comparison between tissue spheroids and cell line spheroids underlines that the tissue-derived breast cancer spheroid model has the advantage of representing the individual patient tumor much more closely in comparison to established breast cancer cell lines and may be more suited for preclinical drug testing.

## Abbreviations

2D: 2 dimensional
3D: 3 dimensional
5-FU: 5-fluorouracil
C: cell lines
Crbp: carboplatin
CO_2_: carbondioxide
C: cyclophosphamide, doxorubicin
ATP: adenosintriphosphate
Cps: counts per second
DMEM: Dulbecco’s modified eagle medium
Doc: docetaxel
EDTA: ethylenediaminetetraacetic acid
ER: estrogen receptor
E: epirubicin
FEC: 5-fluorouracil + epirubicin + cyclophosphamide
HER2: human epidermal growth factor receptor 2
MEM: minimum essential medium
Nd: not done
NEAA: non-essential amino acids
*P*: *p* value
Pac: paclitaxel
PAI1: serpins plasminogen activator inhibitor-1
pCR: pathologic complete response
PIK3CA: phosphatidylinositol-4,5-bisphosphate 3-kinase, catalytic subunit alpha
Ppc: peak plasma concentration
PR: progesterone receptor
T: tissue
uPA: urokinase-type plasminogen activator
